# Highly Efficient Organic/Silicon Hybrid Solar Cells with a MoO_3_ Capping Layer

**DOI:** 10.3390/nano14201630

**Published:** 2024-10-11

**Authors:** Jiahui Chen, Zhangbo Lu, Xiaoting Wang, Yuner Luo, Yun Ma, Gang Lou, Dan Chi, Shihua Huang

**Affiliations:** 1 Key Laboratory of Solid State Optoelectronic Devices of Zhejiang Province, College of Physics and Electronic Information Engineering, Zhejiang Normal University, Jinhua 321004, China; 12345678@zjnu.edu.cn (J.C.); wangxiaoting@sari.ac.cn (X.W.); luoye@sari.ac.cn (Y.L.); mayun@zjnu.cn (Y.M.); lougang@zjnu.edu.cn (G.L.); chidan@zjnu.edu.cn (D.C.); 2 Zhejiang Institute of Photoelectronics, Zhejiang Normal University, Jinhua 321004, China; 3 Zhejiang Institute for Advanced Light Source, Zhejiang Normal University, Jinhua 321004, China

**Keywords:** organic/Si, high work function, MoO_3_ film, built-in potential

## Abstract

Organic/Si hybrid solar cells have attracted considerable attention for their uncomplicated fabrication process and superior device efficiency, making them a promising candidate for sustainable energy applications. However, the efficient collection and separation of charge carriers at the organic/Si heterojunction interface are primarily hindered by the inadequate work function of poly (3,4-ethylenedioxythiophene): poly (styrenesulfonate) (PEDOT:PSS). Here, the application of a high-work-function MoO_3_ film onto the n-Si/PEDOT:PSS surface leads to a notable enhancement in the device’s built-in potential. This enhancement results in the creation of an inversion layer near the n-Si surface and facilitates charge separation at the interface. Simultaneously, it inhibits charge recombination at the heterojunction interface. As a result, the champion PEDOT:PSS/Si solar cell, which incorporates a MoO_3_ interface layer, demonstrates an efficiency of 16.0% and achieves a high fill factor of 80.8%. These findings provide a straightforward and promising strategy for promoting the collection and transmission of charge carriers at the interface of photovoltaic devices.

## 1. Introduction

As of now, c-Si solar cells remain the primary choice in the photovoltaic market because of their exceptional efficiency and durability over time. By utilizing a doped hydrogenated amorphous Si (a-Si:H) layer to passivate Si surfaces, Si heterojunction solar cells have achieved unprecedented efficiencies of approximately 27% [1], positioning them as one of the most promising photovoltaic systems currently available. However, Si heterojunction solar cells continue to experience severe optical and electrical losses due to the inherent parasitic absorption and Auger recombination of doped a-Si:H films [2,3,4]. Furthermore, the preparation of these passive contact layers still necessitates the use of capital-intensive equipment. To address the above issues, novel methods have been developed to replace the doped a-Si:H layer with a hole- or/and electron-selective contact material, and the resulting photovoltaic devices are referred to as doping-free Si solar cells. In recent years, the use of functional materials such as vanadium oxide (VO_X_) [5,6,7], molybdenum oxide (MoO_X_) [8,9,10], and poly (3,4-ethylenedioxythiophene):poly (styrenesulfonate) (PEDOT:PSS) [11,12,13,14,15] as hole transport layers to form hole-selective contact with n-Si has been successfully demonstrated.

The development of PEDOT:PSS/Si hybrid solar cells has received a significant amount of attention as one of the most promising doping-free concept optoelectronic devices, due to its vacuum-free, cryogenic, solution-based fabrication process [16,17,18,19]. The pertinent numerical simulation results illustrate that through the optimization of the structure and interface engineering of PEDOT:PSS/Si photovoltaic devices, a power conversion efficiency (PCE) exceeding 20% can be attained [20,21,22]. Over the past decade, there has been a significant improvement in the performance of PEDOT:PSS/Si hybrid solar cells, with the record PCE being 18.4% [23], indicating substantial potential for further development. However, poor open-circuit voltage (V_OC_) still limits the further improvement of PEDOT:PSS/Si solar cell performance. Recent studies have revealed that the poor V_OC_ is predominantly influenced by the presence of defect states at the heterojunction interface between the n-Si and PEDOT:PSS layers, as well as their impacts on charge extraction and separation [24]. In-depth and systematic investigations have been conducted to tackle the issue of defect states at heterojunction interfaces, leading to the proposal of several effective solutions. For example, by chemically modifying the PEDOT:PSS/Si interface with SiO_X_ [25,26] or 1,1-bis[(di-4-tolylamino)phenyl]cyclohexane [27] films, the performance of photovoltaic devices has been significantly improved. Unfortunately, the V_OC_ of photovoltaic devices is still below 600 mV and satisfactory results are still lacking. It can be observed that the separation of charge carriers at the PEDOT:PSS/Si heterojunction interface is another significant factor that limits the V_OC_. The separation of charge carriers at the interface between PEDOT:PSS and Si is predominantly hindered by the low-work-function PEDOT:PSS layer of the PH1000 type. It is evident that modifying the work function of PEDOT:PSS to increase the band bending level in the junction region has the potential to enhance the built-in potential (V_bi_) and improve the inversion effect, thereby effectively enhancing charge separation at the heterojunction interface.

In this work, a high-work-function molybdenum oxide (MoO_3_) capping layer is deposited on the PEDOT:PSS side of the solar cell device as a functional layer to modify the band offset near the junction region. Through characterization analysis, it is found that the introduction of MoO_3_ thin films significantly increases the V_bi_ of PEDOT:PSS/Si devices, inducing a strong inversion layer on the Si surface, and thereby significantly improving the V_OC_ and PCE of photovoltaic devices. As a result, the champion organic/Si solar cell, based on a MoO_3_ interface layer, exhibits a PCE of 16.0%, with a V_OC_ of 632 mV and a fill factor (FF) as high as 80.8%. These results demonstrate a simple and promising method for efficiently collecting and transmitting interface charges in photovoltaic devices.

## 2. Materials and Methods

### 2.1. Device Fabrication

Single-sided polished 100-oriented n-Si wafers (0.1 Ω·cm) with a thickness of 500 μm were utilized for the preparation of PEDOT:PSS/Si solar cells. Before preparing the photovoltaic devices, Si wafers needed to be pre-cleaned using the standard RCA method. The Si substrates were immersed in a diluted HF solution for 10 min to remove natural oxides, after being cleaned. The PEDOT:PSS (Clevios PH1000) solution was mixed with 5 wt% dimethylsulfoxide (DMSO, Sigma-Aldrich, St. Louis, MO, USA) and 1 wt% Triton-X100 (Sigma Aldrich), and then spin-coated on the polished side of the n-Si, which was followed by thermal annealing at 120 °C. A 500 nm thick Ag top grid electrode was subsequently deposited onto the PEDOT:PSS layer through a shadow mask, and a 200/300 nm full-area Ti/Ag film was deposited on the back of the silicon wafer as the rear electrode. Finally, MoO_3_ films of different thicknesses were deposited on the front surface by thermal evaporation. To avoid any contact issues, the MoO_3_ film does not cover the region where the probe contacts the Ag electrode. The effective area of each solar cell is 0.5 cm^2^.

### 2.2. Device Characterization

The thickness of the MoO_3_ layers were measured by ellipsometry (M-2000DI). The work function of the samples was determined by ultraviolet photoelectron spectroscopy (UPS). The UPS was performed using a Thermo ESCALAB 250XI (Thermo Fisher, Waltham, MA, USA) at the Shiyanjia Laboratory. The current density–voltage (J-V) characteristics of PEDOT:PSS/Si devices were measured by a solar simulator (Sol3A, Newport, Irvine, CA, USA) under AM 1.5 illumination (1000 W m^−2^) standard test conditions. The external quantum efficiency (EQE) spectra were measured in the wavelength range 300–1200 nm with the QEX-10 measuring system. Capacitance and voltage measurements were performed using an Agilent B1500A semiconductor parameter analyzer (Agilent, Santa Clara, CA, USA).

## 3. Results and Discussion

**Figure 1:**
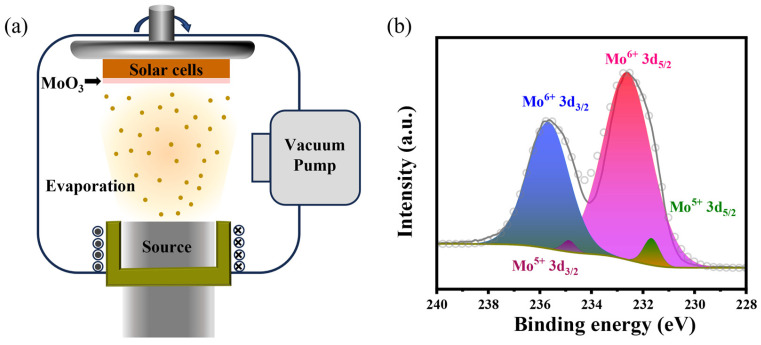
(**a**) Schematic illustration of the thermal evaporation deposition process of the MoO_3_. (**b**) The Mo 3d core level of evaporated MoO_3_ films.

The thermal evaporation of MoO_3_ (Figure 1a) shows significant potential for application in photovoltaic devices. X-ray photoelectron spectroscopy (XPS) measurement was conducted on the MoO_3_ thin films in order to elucidate the composition of the evaporation deposition process. Figure S1 and Figure 1b show the full scan spectra and the XPS spectra of Mo 3d core energy levels for MoO_3_ thin films, respectively. The Mo 3d doublet shows a 3.05 eV spin–orbit splitting between the Mo 3d_3/2_ and 3d_5/2_ bands through simple calculations. In addition, the MoO_3_ film’s Mo 3d spectra were analyzed using two 3d doublets fitted with a Gaussian function to investigate molybdenum in different oxidation states. The Mo 3d_5/2_ peak observed at 232.6 eV and the Mo 3d_3/2_ peak at 235.7 eV suggest the presence of Mo^6+^, while the Mo 3d_5/2_ peak at 231.7 eV and the Mo 3d_3/2_ peak at 234.9 eV are indicative of the existence of Mo^5+^. Moreover, the calculated experimental value of 0.058 based on the Mo^5+^/Mo^6+^ ratio of MoO_3_ can be utilized to further infer that the stoichiometry of Mo:O is 2.97, indicating that the thermal evaporation-prepared MoO_3_ thin film possesses an ideal stoichiometric ratio.

**Figure 2:**
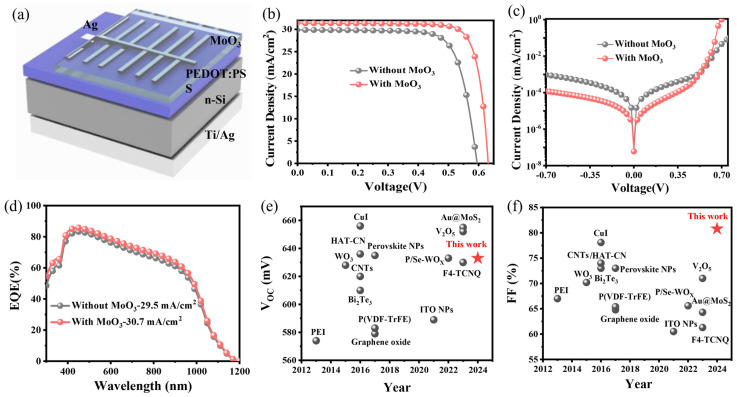
(**a**) Device configuration of PEDOT:PSS/Si solar cells with a MoO_3_ capping layer. (**b**) J–V curves of PEDOT:PSS/Si solar cells without and with the MoO_3_ layer under simulated AM 1.5 illumination at 100 mW cm^−2^. (**c**) Corresponding dark J−V and (**d**) EQE curves of PEDOT:PSS/Si solar cells. The historical (**e**) V_OC_ and (**f**) FF of high-performance planar Si/PEDOT:PSS solar cells with a modified front interface layer are juxtaposed for comparison.

Figure 2a shows the schematic representation of the PEDOT:PSS/Si hybrid solar cell, which includes a MoO_3_ capping layer. We initially deposited MoO_3_ thin films with varying thicknesses onto the PEDOT:PSS layer and systematically investigated the impact of MoO_3_ thickness on device performance. The specific results are shown in Figure S2a and summarized in detail in Table S1. As the thickness of MoO_3_ increases, there are significant increases in V_OC_, short-circuit current density (J_SC_), and FF for devices with MoO_3_ thickness not exceeding 10 nm. However, when the thickness reaches 15 nm, a slight reduction in the photovoltaic parameters of the devices is observed. The impact of MoO_3_ film thickness on J_SC_ is investigated through the analysis of reflectance spectra, as shown in Figure S2b. It is evident that MoO_3_ films with a thickness below 15 nm exhibit reduced reflectance across the entire spectral region. When the thickness of MoO_3_ increases to 30 nm, although the reflectance further decreases in the wavelength range of 600 to 1100 nm, it significantly increases in the ultraviolet wavelength range. Therefore, achieving an optimal thickness of MoO_3_ is essential for enhancing the J_SC_ of the device. By comparison, it is found that a 10 nm thick MoO_3_ layer can achieve the optimal photovoltaic performance of the PEDOT:PSS/Si solar cell. Unless otherwise specified, the thickness of MoO_3_ is 10 nm.

Figure 2b shows the J-V curves of PEDOT:PSS/Si solar cells without and with the MoO_3_ layer, and the corresponding photovoltaic parameters are summarized in Table 1. The Ag/PEDOT:PSS/n-Si/Ti/Ag structure-based device achieves a PCE of 13.4%, with V_OC_, J_SC_, and FF values of 595 mV, 29.8 mA/cm^2^, and 75.4%, respectively. After being coated with a MoO_3_ layer, the device exhibits superior performance, with a V_OC_ of 632 mV, a J_SC_ of 31.4 mA/cm^2^, an FF of 80.8%, and a PCE of 16.0%. For the purpose of comparison, we have provided a summary of the V_OC_ and FF values for Planar Si/PEDOT:PSS solar cells with front interface modification layers, as reported in the literature (Figure 2e,f and Table S2). Compared to other high-performance PEDOT:PSS/Si solar cells, the devices modified with MoO_3_ have demonstrated superior photovoltaic parameters and performance. It is noteworthy that the FF of the MoO_3_-based photovoltaic devices in this study represents the highest reported value in PEDOT:PSS/Si solar cells to date.

The improvement of photovoltaic parameters of the device is closely related to the improvement of junction quality. Figure 2c shows the dark J−V characteristics of the PEDOT:PSS/Si solar cells without and with the MoO_3_ layer. The internal parameters of the device (Table S3) are obtained by fitting the lnI-V curve (Figure S3) and Equation (1).

(1) $$I=I_{0}\left[exp\left(\frac{ev}{nkT}\right)-1\right]={\mathrm{AA}}^{*}T^{2}exp\left(-\frac{\emptyset_{b}}{kT}\right)\left[\left(\frac{eV}{nkT}\right)\right]-1$$

(2) $$V_{OC}=\frac{kT}{q}ln\left(\frac{I_{SC}}{I_{0}}\right)+1$$

where n is the ideal factor, I is the current value, I_0_ is the saturation current, V is the applied voltage, q is the electronic charge, A is the contact area (0.5 cm^2^), *T* is the absolute temperature (298 K), k is the Boltzmann constant, and A* is the effective Richardson constant (~252 A cm^−2^ K^−2^ for n-Si). After conducting calculations, it can be determined that incorporating a MoO_3_ layer leads to a significant decrease in the n values of PEDOT:PSS/Si devices from 2.39 to 1.66, demonstrating a noticeable enhancement in the quality of the heterojunction. In the absence of a MoO_3_ layer, the I_0_ of PEDOT:PSS/Si devices is measured at 2.76 × 10^−7^ A, which decreases to 5.75 × 10^−9^ A with the introduction of a MoO_3_ layer. The relationship between V_OC_ and I_0_ can be described by Equation (2), indicating that the reduction in I_0_ leads to an increase in the V_OC_ value of the device. The increase in V_OC_ of PEDOT:PSS/Si devices is ascribed to the incorporation of a MoO_3_ layer, resulting in the augmentation of V_bi_ near the heterojunction region and improved field-effect passivation, which will be further elucidated later. Additionally, with the incorporation of the MoO_3_ layer, the barrier height (Φ_B_) of the device is raised from 0.81 to 0.91 eV. The EQE spectra of devices without and with the MoO_3_ layer are shown in Figure 2d. The device, which has a layer modified by MoO_3_, shows an improved optical response in the range of 300 to 1200 nm. This leads to a J_SC_ of 29.5 mA/cm^2^ for the control solar cells and 30.7 mA/cm^2^ for the MoO_3_-modified solar cells, consistent with the results obtained from the J−V curve and photovoltaic parameters.

**Figure 3:**
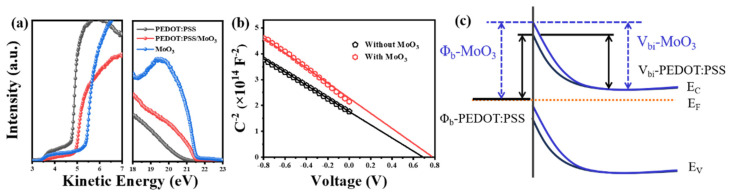
(**a**) UPS measured work function of the as-prepared PEDOT:PSS, PEDOT:PSS/MoO_3_, and MoO_3_ films. (**b**) Capacitance−voltage measurements of the Si/PEDOT:PSS heterojunction solar cells without and with the thin layer of MoO_3_. (**c**) Energy band diagram of the PEDOT:PSS/Si hybrid solar cells.

To elucidate the effect of the MoO_3_ layer on the interface of PEDOT:PSS/Si heterojunction, the electronic structures of PEDOT:PSS, PEDOT:PSS/MoO_3_, and MoO_3_ are determined through UPS measurements (Figure 3a). The work functions of pristine PEDOT:PSS and MoO_3_ films are 4.8 and 5.4 eV, respectively. When MoO_3_ is deposited on PEDOT:PSS thin films, the work function of PEDOT:PSS increases by 0.2 eV. The enhanced work function of the PEDOT:PSS film may induce stronger inversion effects, leading to higher V_bi_ in the Si/PEDOT:PSS heterojunction. The V_bi_ of the corresponding device can be fitted from the 1/C^2^-V curve (Figure 3b) and extracted by means of Equation (3).

(3) $$\frac{1}{C^{2}}=\frac{2(V_{bi}-V)}{q\varepsilon N_{d}}$$

where N_d_ is the Si doping level, ε is Si permittivity, and V is applied bias voltage. The V_bi_ of PEDOT:PSS/Si solar cells with and without the MoO_3_ layer are 0.78 and 0.68 V, respectively. Fortunately, the introduction of the MoO_3_ layer significantly increased the V_bi_ value of the device by 0.1 V. Figure 3c shows the calculated energy band diagram of PEDOT:PSS/Si hybrid solar cells with and without MoO_3_ capping layer. A large V_bi_ typically leads to band bending near the Si surface, which is responsible for the increased V_OC_ values in devices with a MoO_3_ layer.

**Figure 4:**
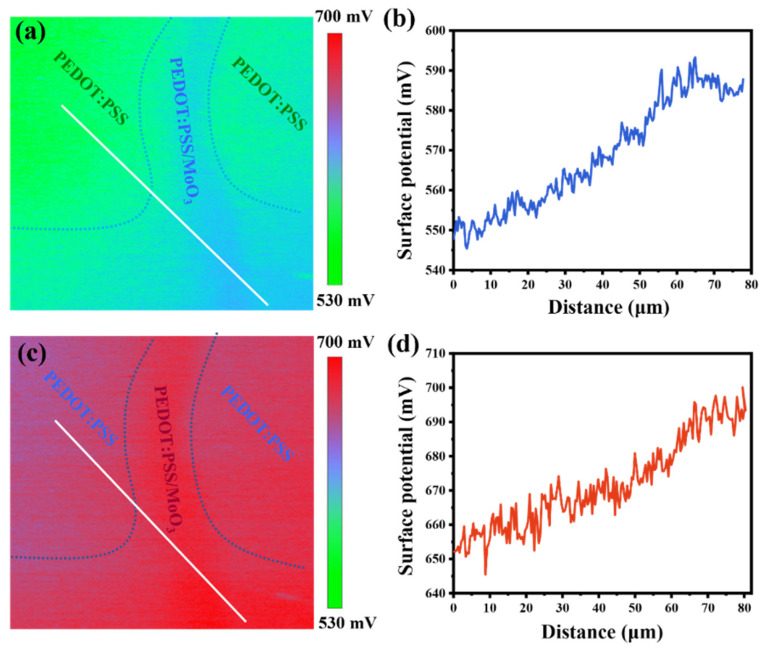
Surface potential image of MoO_3_ film partially covering the surface of PEDOT:PSS (**a**) without and (**c**) with solar irradiation. Both of the scale bars are 10 µm. Cross-sectional line profile of the surface potential image (**b**) without and (**d**) with solar irradiation.

To further validate the enhanced carrier transfer or separation at the heterojunction interface due to the introduction of the MoO_3_ layer, we conducted surface-potential measurements of Si/PEDOT:PSS and Si/PEDOT:PSS/MoO_3_ films in both dark and illuminated conditions using Kelvin probe force microscopy (KPFM), as depicted in Figure 4. The surface potential of the MoO_3_ layer is enhanced by 40 mV under dark conditions and by 50 mV under irradiation conditions, compared to the original PEDOT:PSS layer. The observed rise in surface potential provides additional evidence for the heightened work function of the PEDOT:PSS layer following its coating with a MoO_3_ layer. The enhanced work function leads to a more robust built-in electric field, thereby facilitating an increased injection of holes from n-Si into PEDOT:PSS. Furthermore, the significant variation in surface potential difference observed under dark and irradiation conditions can be attributed to the pronounced photoelectric effect that occurs within the n-Si/PEDOT:PSS heterostructure coated with MoO_3_ when exposed to light.

**Figure 5:**
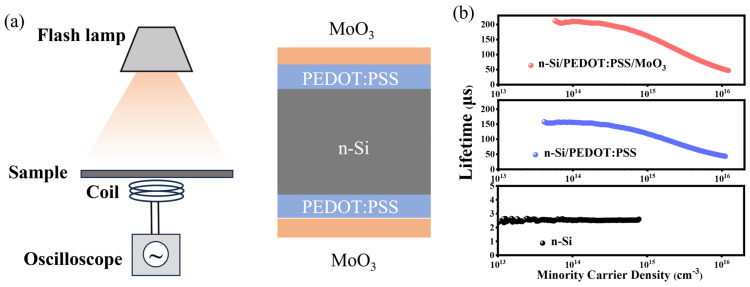
Characterization of effective carrier lifetime. (**a**) The schematic of the lifetime measurements using a Sinton Instruments WCT-120 photo-conductance tool and lifetime test structure. (**b**) The measured effective carrier lifetime as a function of excess carrier density for n-type crystalline silicon wafers symmetrically passivated without and with MoO_3_ films.

The introduction of the MoO_3_ interface layer also provides excellent field-effect passivation at the n-Si/PEDOT:PSS interface, effectively suppressing carrier recombination at the heterojunction interface. To investigate the impact of MoO_3_ on surface recombination at the PEDOT:PSS/Si heterojunction, minority carrier lifetime (τ_eff_) tests were performed on the relevant sample surfaces using quasi-steady-state photoconductivity (QSSPC) technology (Figure 5a). Figure 5b shows the τ_eff_ versus the excess carrier density (Δn) for n-Si substrates passivated by PEDOT:PSS and PEDOT:PSS/MoO_3_ films. The τ_eff_ of an n-type silicon wafer is 3 µs at an injection level of 10^14^ cm^−3^. Under the same Δn conditions, the τ_eff_ of Si/PEDOT:PSS and Si/PEDOT:PSS/MoO_3_ samples reaches 157 and 209 µs, respectively. This result indicates that the introduction of a MoO_3_ layer significantly increases the τ_eff_ of the photovoltaic device. There is a close relationship between minority carrier lifetime and surface recombination rate, as shown in Equation (4).

(4) $$\frac{1}{\tau_{eff}}=\frac{1}{\tau_{bulk}}+\frac{2S}{W}$$

where τ_bulk_ is the bulk recombination lifetime, S is the surface recombination rate, and W is the wafer thickness. Due to the use of the same silicon wafer, the τ_bulk_ is fixed. Therefore, the surface recombination rate is inversely proportional to the minority carrier lifetime. The introduction of a MoO_3_ capping layer can be seen to significantly reduce the defect recombination rate at the heterojunction interface, resulting in a higher minority carrier lifetime.

## 4. Conclusions

In conclusion, a high-work-function MoO_3_ capping layer was deposited on the PEDOT:PSS side of a solar cell device as a functional layer to modify the band offset near the junction region. Characterization analysis reveals that the introduction of MoO_3_ thin films significantly increases the V_bi_ of PEDOT:PSS/Si devices, inducing a strong inversion layer on the Si surface, and significantly improving the V_OC_ and PCE of photovoltaic devices. Therefore, the champion organic/Si solar cell, based on a MoO_3_ interface layer, exhibits a PCE of 16.0%, with a V_OC_ of 632 mV and an FF as high as 80.8%. This provides a straightforward and promising approach to enhancing the collection and transmission of charge carriers at the interface of photovoltaic devices.

## Figures and Tables

**Table 1:** The photovoltaic parameters of the hybrid solar cells with and without the MoO_3_ layer.

Device	*V_OC_* (mV)	*Jsc* (mA/cm^2^)	FF (%)	PCE (%)	*Rs* (Ω·cm^2^)
w/o MoO_3_	595	29.8	75.4	13.4	4.5
	(593 ± 6)	(29.4 ± 0.5)	(75.1 ± 0.9)	(13.1 ± 0.3)	(4.7 ± 0.6)
w/MoO_3_	632	31.4	80.8	16.0	2.5
	(631 ± 2)	(30.4 ± 0.5)	(80.0 ± 0.4)	(15.3 ± 0.4)	(2.9 ± 0.3)

## Data Availability

Data are contained within the article and Supplementary Materials.

## Supplementary Materials

The following supporting information can be downloaded at: https://www.mdpi.com/article/10.3390/nano14201630/s1, Figure S1: Full-scan XPS spectra of thin films composed of MoO_3_. Figure S2: (a) J-V curves of PEDOT:PSS/Si solar cells with different thicknesses of MoO_3_ thin layers under simulated AM 1.5 illumination at 100 mW cm^−2^. (b) Reflectance spectra of polished n-Si and n-Si/PEDOT:PSS/MoO_3_ surfaces. Figure S3: n and I_0_ values extrapolated from dln(I) vs. voltage curves. Table S1: The photovoltaic parameters of the hybrid solar cells with different thicknesses of MoO_3_ thin layers. Table S2: Previous reports on high-performance planar Si/PEDOT:PSS solar cells with a front interface modification layer. Table S3: Summary of n, I_0_ and Φ_B_, which is extrapolated from dlnI vs. voltage curves. Refs. [26,27,28,29,30,31,32,33,34,35,36,37,38,39] are cited in the Supplementary Materials.
