# Supplementary material for: Highly Efficient Organic/Silicon Hybrid Solar Cells with a MoO3 Capping Layer
Source: Nanomaterials (Basel). 2024 Oct 11;14(20):1630. doi: 10.3390/nano14201630 (PMC11510610; doi:10.3390/nano14201630)
Supplement: Supplementary file 1 [file nanomaterials-14-01630-s001.zip › nanomaterials-3229733-supplementary.pdf]

Supporting Information

Highly efficient organic/silicon hybrid solar cells with a MoO_3 capping layer

Jiahui Chen [1], Zhangbo Lu [1,2,3] *, Xiaoting Wang [1], Yuner Luo [1], Yun Ma [1], Gang Lou [1], Dan Chi [1], Shihua, Huang [1, *]

[1] Key Laboratory of Solid State Optoelectronic Devices of Zhejiang Province, College of Physics and Electronic Information Engineering, Zhejiang Normal University, Jinhua 321004, China;

[2] Zhejiang Institute of Photoelectronics, Zhejiang Normal University, Jinhua, 321004, China;

[3] Zhejiang Institute for Advanced Light Source, Zhejiang Normal University, Jinhua, 321004, China

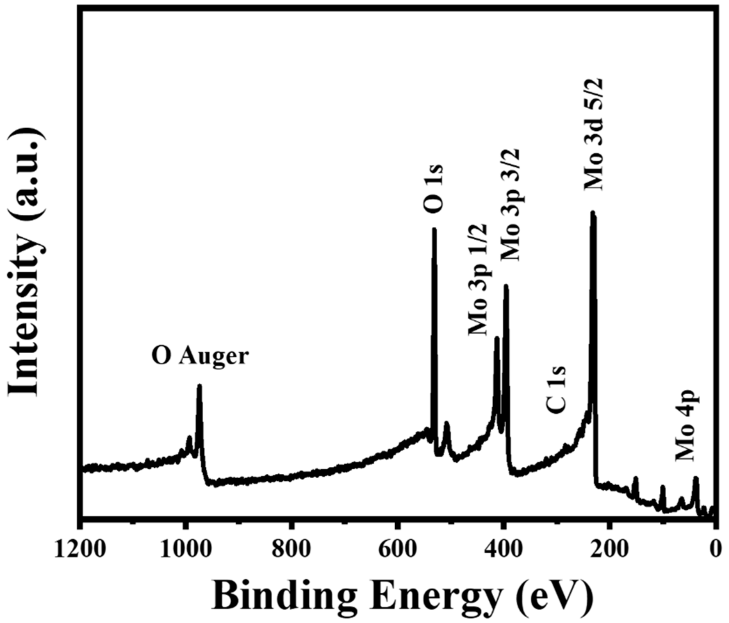

Figure S1. Full-scan XPS spectra of thin films composed of MoO_3.

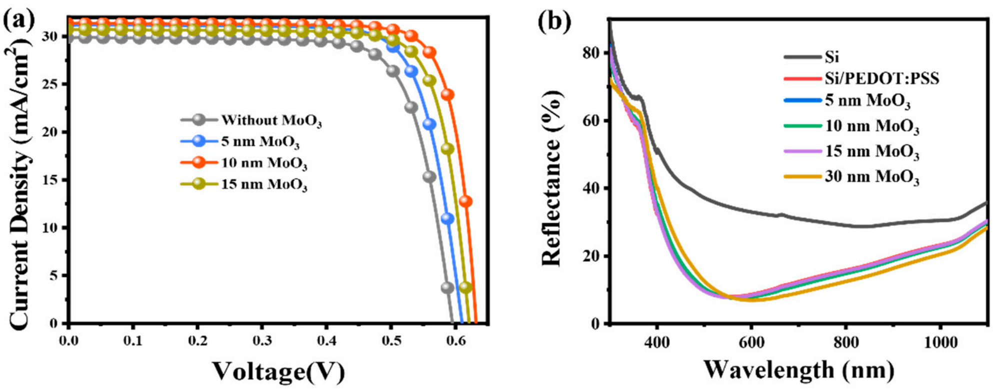

Figure S2. (a) J–V curves of PEDOT:PSS/Si solar cells with different thicknesses of MoO_3 thin layers under simulated AM 1.5 illumination at 100 mW cm^{-2}. (b) Reflectance spectra of polished n-Si and n-Si/PEDOT:PSS/MoO_3 surfaces.

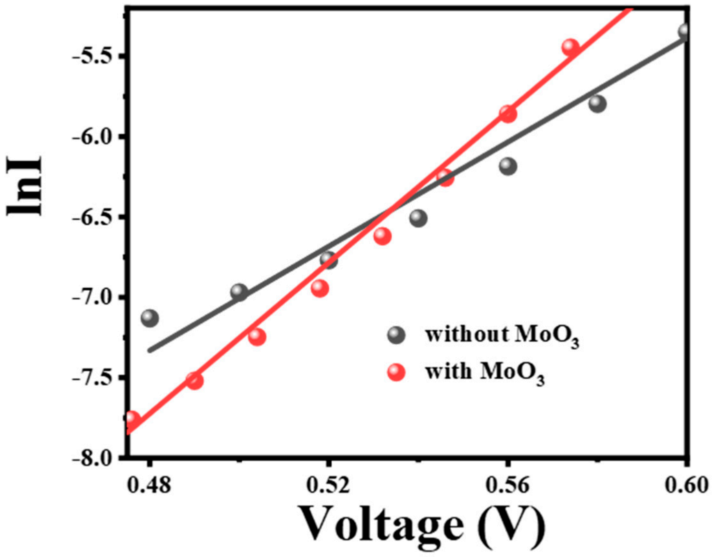

Figure S3. diode ideality factor (n) and reverse saturation current (I_0) values extrapolated from dln(I) vs voltage curves.

Table S1. The photovoltaic parameters of the hybrid solar cells with different thicknesses of MoO_3 thin layers.

MoO_3 Thickness (nm)	V_{oc} (mV)	J_{sc} (mA/cm^2)	FF (%)	PCE (%)
0	595	29.8	75.4	13.4
5	610	31.1	76.5	14.6
10	632	31.4	80.8	16.0
15	621	30.7	79.3	15.1

Table S2. Previous reports on high-performance planar Si/PEDOT:PSS solar cells with a front interface modification layer.

Front interface	V_{oc} (mV)	J_{sc} (mA/cm^2)	FF (%)	PCE (%)	Year	Ref.
Graphene oxide	579	31.9	64.8	12.0	2017	[1]
PEI	574	28.6	67.0	11.0	2013	[2]
ITO NPs	589	33.6	60.5	12.0	2021	[3]
CNTs	620	26.4	74.0	12.1	2016	[4]
WO_3	628	26.4	70.2	11.7	2015	[5]
P(VDF-TrFE)	583	30.8	65.4	11.7	2017	[6]
Bi_2Te_3	610	27.1	73.0	12.1	2016	[7]
Perovskite NPs	635	30.8	73.0	14.3	2017	[8]
HAT-CN	636	28.8	74.0	13.4	2016	[9]
CuI	656	28.0	78.1	14.3	2016	[10]
P/Se-WO_X	633	33.5	65.6	13.6	2022	[11]
F4-TCNQ	630	33.3	61.3	13.2	2023	[12]
Au@MoS_2	655	33.2	64.3	14.0	2023	[13]
V_2O_5	652	32.8	71.0	15.2	2023	[14]
MoO_3	633	31.4	80.8	16.0	2024	This work

Table S3. Summary of n, I_0 and barrier height (Φ_B), which is extrapolated from dlnI vs voltage curves.

Device	n	I_0 (A)	Φ_B (eV)
w/o MoO_3	2.39	2.76×10^{-7}	0.81
w/MoO_3	1.66	5.75×10^{-9}	0.91

References

1. Jiang, X.; Wang, Z.; Han, W.; Liu, Q.; Lu, S.; Wen, Y.; Hou, J.; Huang, F.; Peng, S.; He, D.; et al. High performance silicon–organic hybrid solar cells via improving conductivity of PEDOT:PSS with reduced graphene oxide. *Appl. Surf. Sci.* **2017,** *407* 398-404.

2. Liu, D.; Zhang, Y.; Fang, X.; Zhang, F.; Song, T.; Sun, B. An 11%-Power-Conversion-Efficiency Organic–Inorganic Hybrid Solar Cell Achieved by Facile Organic Passivation. *IEEE Electron Device Lett.* **2013**, *34*, 345-347.

3. Zhao, Y.; Zhang, L.; Lv, M.; Jiao, C.; Cheng, P.; Fu, Y.; Li, J.; Liu, Q.; He, D. Improvement of the Optoelectrical Properties of a Transparent Conductive Polymer via the Introduction of ITO Nanoparticles and Its Application in Crystalline Silicon/Organic Heterojunction Solar Cells. *ACS Appl. Mater. Interfaces* **2021**, *13*, 31171-31179.

4. Duan, X.; Han, J.; Xia, Z.; Song, T.; Li, Q.; Li, H.; Sun, B. Semiconducting single-walled carbon nanotubes as interfacial modification layers for organic-Si solar cells. *Org. Electron.* **2016**, *28*, 205-209.

5. Mu, X.; Yu, X.; Xu, D.; Shen, X.; Xia, Z.; He, H.; Zhu, H.; Xie, J.; Sun, B.; Yang, D. High efficiency organic/silicon hybrid solar cells with doping-free selective emitter structure induced by a WO_3 thin interlayer. *Nano Energy* **2015**, *16*, 54-61.

6. Kang, S. B.; Jeong, M. H.; Choi, I. Y.; Sohn, S.-D.; Kim, S. H.; Shin, H.-J.; Park, W. I.; Shin, J. C.; Song, M. H.; Choi, K. J. Self-assembled, highly crystalline porous ferroelectric poly(vinylidene fluoride-co-trifluoroethylene) interlayer for Si/organic

hybrid solar cells. *Nano Energy* **2017**, *41*, 243-250.

7. Zhao, M.; Zhang, J.; Gao, N.; Song, P.; Bosman, M.; Peng, B.; Sun, B.; Qiu, C. W.; Xu, Q. H.; Bao, Q.; et al. Actively Tunable Visible Surface Plasmons in Bi_2Te_3 and their Energy-Harvesting Applications. *Adv. Mater.* **2016**, *28*, 3138-3144.

8. Wang, Y.; Xia, Z.; Liu, L.; Xu, W.; Yuan, Z.; Zhang, Y.; Sirringhaus, H.; Lifshitz, Y.; Lee, S. T.; Bao, Q.; et al. The Light-Induced Field-Effect Solar Cell Concept – Perovskite Nanoparticle Coating Introduces Polarization Enhancing Silicon Cell Efficiency. *Adv. Mater.* **2017**, *29*, 1606370.

9. Liu, Y.; Zhang, Z.-g.; Xia, Z.; Zhang, J.; Liu, Y.; Liang, F.; Li, Y.; Song, T.; Yu, X.; Lee, S.-t.; et al. High Performance Nanostructured Silicon–Organic Quasi p–n Junction Solar Cells via Low-Temperature Deposited Hole and Electron Selective Layer. *ACS Nano* **2015**, *10*, 704-712.

10. He, J.; Gao, P.; Ling, Z.; Ding, L.; Yang, Z.; Ye, J.; Cui, Y. High-Efficiency Silicon/Organic Heterojunction Solar Cells with Improved Junction Quality and Interface Passivation. *ACS Nano* **2016**, *10*, 11525-11531.

11. Ni, Z.; Ding, S.; Zhang, H.; Dai, R.; Chen, A.; Wang, R.; Zhang, J.; Zhou, Y.; Yang, J.; Sun, T.; et al. Phosphorus and Selenium Co-Doped WO_3 Nanoparticles for Interface Modification and Photovoltaic Properties Enhancement of Monolayer Planar Silicon/PEDOT:PSS Hybrid Solar Cells. *Adv. Mater. Interfaces* **2022**, *9*, 2200812.

12. Yang, C.; Luo, Z.; Ma, W.; Li, S.; Lv, G.; Fu, K.; Liu, K.; Li, H.; Sun, H.; Chen, X. Study on the Fabrication of PEDOT:PSS/Si Hybrid Solar Cells Incorporated with F4TCNQ and VTMO. *J. Phys. Chem. C* **2023**, *127*, 7974-7986.

13. Wang, J.; Zhou, W.; Wei, Q.; Liu, G.; Yuan, X.; Pen, H.; Zhang, G.; Wang, R.; Wang, C.; Yang, Y. Effect of Au@MoS_2 Contacted PEDOT:PSS on Work Function of Planar Silicon Hybrid Solar Cells. *Adv. Mater. Interfaces* **2023**, *10*, 2300187.

14. Luo, Z.; Yang, C.; Chen, X.; Ma, W.; Li, S.; Fu, K. Improving open-circuit voltage and short-circuit current of high-efficiency silicon-based planar heterojunction solar cells by combining V_2O_5 with PEDOT:PSS. *J. Materiomics* **2023**, *9*, 438-446.
